# Correlation of the degree of clavicle shortening after non-surgical treatment of midshaft fractures with upper limb function

**DOI:** 10.1186/s12891-015-0585-3

**Published:** 2015-06-17

**Authors:** Gustavo Santiago de Lima Figueiredo, Marcel Jun Sugawara Tamaoki, Bruno Dragone, Artur Yudi Utino, Nicola Archetti Netto, Marcelo Hide Matsumoto, Fábio Teruo Matsunaga

**Affiliations:** Orthopaedics and Traumatology Department, São Paulo Federal University, Napoleão de Barros Street 715, São Paulo, Brasil

**Keywords:** Fracture, Clavicle, Shortening, Conservative treatment, DASH

## Abstract

**Background:**

Despite the use of non-surgical methods to treat for the majority of midshaft fractures of the clavicle, it is remains controversial whether shortening of this bone following non-surgical treatment of a middle third fracture affects upper limb function.

**Methods:**

We conducted a cohort study by sequentially recruiting 59 patients with a fracture of the middle third of the clavicle. All patients were treated nonsurgically with a figure-of-eight bandage until clinical and radiological findings indicated healing of the fracture. Functional outcome was assessed using the Disability of Arm, Hand and Shoulder (DASH) score revalidated for the Portuguese language, other outcomes assessed included: pain measured by visual analogue scale (VAS); radiographies to measure the degree of shortening, fracture consolidation and fracture malunion. Information were also collected regarding the mechanism of injury, patient’s daily activities level and epidemiological features of the patient cohort. The results of our findings are expressed as the comparison of the functional outcome with the degree of shortening.

**Results:**

Patients were assessed six weeks and one year after injury. In the first evaluation, the mean DASH score was 28.84 and pain measured by VAS was 2.57. In the second evaluation (one year after injury) the mean DASH score was 8.18 and pain was 0.84. The mean clavicle shortening was 0.92 cm, ranging from 0 to 3 cm (SD = 0.64). There were no correlation between the degree of shortening and DASH score after six weeks and one year (p = 0.073 and 0.706, respectively). When only patients with of shortening greater than 2 cm were assessed for correlation, the result did not change.

**Conclusion:**

We conclude that clavicle shortening after nonsurgical treatment with a figure-of-eight bandage does not affect limb function, even when shortening exceeds 2 cm.

**Trial registration:**

ISRCTN85206617. Registered 12 May 2014

## Background

Fractures of the clavicle are very common, representing approximately 2.6 % of all skeletal fractures^1^, where fracture of the middle third of the clavicle represents for 80 % to 85 % of clavicle fractures. Anatomically, the middle third of the clavicle is the narrowest portion of the bone and is less coated with soft tissues, making this portion of the bone more susceptible to fractures [1–6]. Very often this type of fracture is associated with displacement caused by muscle insertions: the sternocleidomastoid muscle pulls the medial fragment upward and posteriorly, and the pectoralis major muscle, the deltoid muscle, and gravity pull the lateral fragment downward and anteriorly [7].

Nonsurgical treatment of clavicle fractures with a figure-of-eight bandage or sling have been used for decades with excellent results and low complication rates [8–10]. However, some recent studies have questioned these results, especially in cases of displacement and clavicular shortening [11, 12].

The clavicle is the only bone that connects the shoulder to the axial skeleton. Shortening of the clavicle, according to anatomical studies, is associated to decreased strength and range of motion [13]. Other studies have also demonstrated a relationship between shortening and worse functional outcomes, recommending surgical treatment in case where shortening is greater than 2 cm [14].

In contrast, retrospective studies report good functional outcome and low complication rates in patients that have undergone conservative treatment even when the clavicle is shortened [10, 15]. Similarly, the congenital absence of the clavicle (e.g., Cleidocranial dysostosis) or its removal as part of surgical procedures (e.g., Mumford surgery, vascular surgery) has little influence on upper limb function in these patients [16].

Thus, it is still controversial whether clavicle shortening affects upper limb function. In view of this controversy, we developed this study to assess the relationship between shortening of the clavicle after conservative treatment with figure-of-eight bandage and upper limb function. Our null hypothesis is that there is no relationship between shortening and functional impairment.

## Methods

This cohort study included 59 sequentially recruited patients with midshaft clavicle fractures. They were treated and assessed in the Discipline of Hand and Upper Limb Surgery at Universidade Federal de Sao Paulo (UNIFESP) from January 2010 to June 2012.

We included patients aged 18 and older with a fracture of the middle third of the clavicle by clinical examination and radiographies. Exclusion criteria included neurological and vascular associated injuries, open fractures, associated fracture in the upper limb, bilateral fractures, clavicle fractures with bone contact (assessed by anteroposterior and Zanca radiographic views), “fractures with 14 or more days old since fracture, previous surgery, in the affected limb or previous disease that could change outcomes.

All patients were informed about the objectives of the protocol and agreed and signed the Consent Form to participate in the study. This project was approved by the National Ethics Committee on Research under the number 11376613.2.0000.5505.

**Fig. 1:**
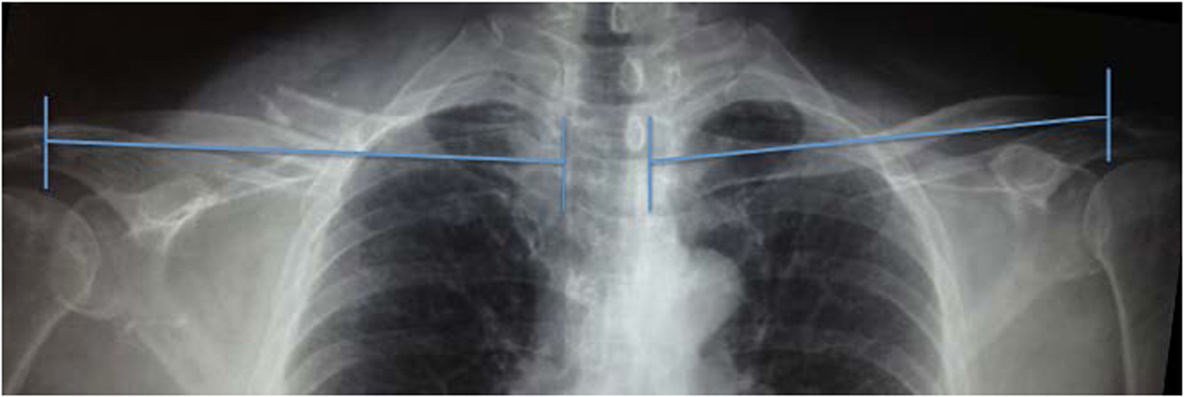
Measurement of shortening

All patients were treated with a figure-of-eight bandage, for a minimum of six weeks until clinical and radiological healing of the fracture were observed. In the first evaluation, the length of both clavicles was measured on a single anteroposterior radiograph with the patient seated [22]. Both clavicles of each patient were measured from the centre of the sternoclavicular joint to the centre of the acromioclavicular joint; the degree of shortening was calculated as the difference between the lengths of the two clavicles (Fig. 1).

During treatment, patients were allowed to use the affected limb as tolerated. Each patient underwent rehabilitation from the sixth week onward, with exercises to increase range of motion and passive, active, and progressive strengthening.

The main outcome measured was using the Disability of Arm, Hand and Shoulder (DASH) score revalidated for the Portuguese language [24], consisting of 30 questions concerning the level of difficulty in completing everyday tasks, and pain was assessed using the visual analogue scale (VAS). Both outcomes were assessed at consultations six weeks and one year after injury. Assessment at 6 weeks aimed to evaluate the early outcome, especially in relation to pain. The evaluation after 1 year aimed to evaluate the late outcome, especially in relation to function. We also evaluated demographics and epidemiological characteristics of the cohort.

Statistical analysis were performed by comparing the results of the DASH questionnaire and pain level of patients with the degree of clavicle shortening on the affected side using the Spearman correlation. Patient functional outcomes were also compared with patient epidemiological characteristics using the Mann Whitney test.

The main outcomes assessed for correlation with clavicle shortening were pain levels and limb function. Second, we examined the association of the objective variables age, sex, and affected limb with the dichotomous, subjective variables of occupation, cause of trauma, aesthetic satisfaction, and occurrence of complications.

## Results

Seventy patients were seen during the study period. After the initial evaluation 11 patients were within the exclusion criteria: two open fractures, one ipsilateral humeral fracture, five fractures with bone contact, two fractures met with more than 14 days of the initial trauma and one patient with contralateral cuff injury, totaling 59 patients included.

Of the fifty nine patients included in the protocol, 48 were males (81.4 %) and 11 females (18.6 %). The mean age was 34 years, ranging from 17 to 64 years (SD = 12.73). The dominant limb was affected in 27 of patients (45.76 %) and the left side accounted for 34 (57.6 %) of the fractures (Table 1).

**Table 1:** Description of epidemiologic results

Variable	Frequency	%
Gender		
Male	48	81.4
Female	11	18.6
Ethnicity		
Caucasian	52	88.1
Other	7	11.9
Dominant limb		
Right	57	96.6
Left	2	3.4
Affected limb		
Right	25	42.4
Left	34	57.6
Mechanism of injury		
High energy	42	71.2
Low energy	17	28.8
Occupation		
High demand	24	40.7
Low demand	35	59.3
Total	59	100

The inclusion of patients in the protocol was performed 1 to 14 days after the injury, with a mean of 6.56 days (SD = 3.77 days). All patients were followed for at least one year, with a loss of follow up of 5 patients (8.47 %) (5 patients) (Table 2).

**Table 2:** Description of DASH, VAS, shortening and age result

Variable	Mean	SD	Median	Minimum	Maximum	N
Age (years)	34	12.73	30.37	17.91	64.21	59
DASH 6 weeks	28.84	23.62	28.33	0.83	85.83	55
DASH 1 year	3.38	9.21	0.00	0.00	58.00	54
VAS 6 weeks	2.57	2.52	1.80	0.00	9.50	54
VAS 1 year	0.34	0.98	0.00	0.00	5.00	54
Shortening (cm)	0.92	0.64	0.80	0.00	3.00	54

The functional outcome assessed by the DASH questionnaire at six weeks and one year averaged 28.84 and 8.18, respectively. Pain level assessed by VAS at six weeks and one year averaged 2.57 and 0.84 respectively.

**Fig. 2:**
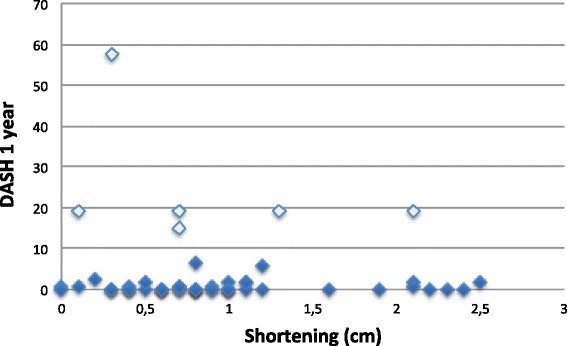
Correlation of DASH results in 1 year and shortening (cm)

The degree of shortening averaged 0.92 cm, ranging from 0 to 3 cm (SD = 0.64 cm). There was no correlation between the shortening of the limb and the DASH score of function at six weeks or one year (p = 0.073 and 0.706 respectively). Setting a minimum threshold of 2 cm shortening did not improve the correlation (Tables 3 and 4) (Fig. 2).

**Table 3:** Corralation of DASH and VAS with shortening

Shortening correlation			
Variable	Correlation	N	p
DASH 6 weeks	−0.246	54	0.073
DASH 1 year	−0.017	54	0.904
VAS 6 weeks	−0.078	54	0.577
VAS 1 year	0.002	54	0.991

**Table 4:** Correlation of DASH and VAS with shortening when set a threshold of 2 cm

Variable	Shortening	Mean	CI	Minimum	Maximum	N	p
DASH 6 weeks	<2 cm	29.27	23.56	0.83	85.83	47	0.705
	≥2 cm	25.66	27.42	0.83	83	7	
DASH 1 year	<2 cm	3.38	9.56	0	58	47	0.528
	≥2 cm	3.33	7.02	0	19	7	
VAS 6 weeks	<2 cm	2.62	2.61	0	9.5	47	>0.999
	≥2 cm	2.24	1.96	0.1	4.8	7	
VAS 1 year	<2 cm	0.37	1.04	0	5	47	0.782
	≥2 cm	0.16	0.42	0	1	7

**Table 5:** Correlation of DASH with mechanism of injury

Variable	Mechanism of injury	Mean	CS	Median	Minimum	Maximum	N	p
DASH 6 weeks	High energy	28.41	22.71	29.165	0.83	85.83	40	0.940
	Low energy	30.00	26.70	17.5	0.83	85.83	15	
DASH 1 year	High energy	3.50	10.26	0	0	58	39	0.629
	Low energy	3.05	5.95	0	0	19	15

Seven patients with shortening greater than 2 cm scored lower on the VAS than patients whose shortening was less than 2 cm (47 patients) (0.16 compared to 0.37). This suggests that patients with a greater degree of shortening tended to experience less pain, although this difference was not significant (p = 0.782).

The mechanisms of injury were divided in either high or low energy trauma, and the patient occupations were classified as high or low demand. High-energy trauma accounted for 42 (71.2 %) of all fractures. Of these high-energy trauma injuries, 34 (80.95 %) resulted from motorcycle accidents, which caused more injuries than any other trauma [17].

The DASH score in cases of high-energy trauma averaged 3.50 with a SD of 10.26, while the score in cases of low-energy trauma averaged 3.05 with a SD = 5.95. However, this difference was not significant (p = 0.629).

After one year of follow-up and return to their occupation, patients with high-demand occupations such as mason or woodworker had the best average DASH score: 2.91 (SD = 5.93). However, the mean score was not significantly different from the score of patients with low-demand occupation such as teacher or salesman, 3.75 (SD = 11.26).

Ten patients (16.6 %) presented a complication. Six patients (11.1 %) developed non-union after nine months of treatment, [19] all of them had less than 1cm of shortening. One patient(1.85 %) presented transient paraesthesia around the fracture. And three patients (5.55 %) demonstrated aesthetic dissatisfaction with osseous deformity.

For all patients who developed non-union, surgical treatment was indicated, but they all, but they chose not to undergo surgery because they were satisfied with the function of the limb.

## Discussion

Classically, most mid-third diaphyseal fractures of the clavicle are treated using a non-surgical figure-of-eight bandage or a simple sling. However, some authors have recently questioned this type of treatment in certain types of fracture, particularly those with large deviations due to non-union rates higher than those described in literature [8, 9] and functional deficit in the affected limb [14].

The consequences of malunion of the clavicle are still controversial. According to some authors [11, 14, 18], shortening greater than 1,5 – 2,0 cm is associated with worse functional outcome. In contrast, others have demonstrated no direct relationship between the degree of shortening and function [10, 15]; this result is also demonstrated in our study, in which we observed seven patients with clavicular shortening greater than 20 mm. All seven exhibited clinical outcomes at one year classified as excellent. Their mean DASH score of 3.33 was similar to the mean DASH score of 3.38 for patients with less shortening.

Studies that demonstrate a direct relationship between the shortening and loss of function in the limb are generally retrospective, whereas prospective studies such as present study do not show this result. The differences in findings may result from factors such as selection bias; patients with longer follow-up periods tend to be those with the worst outcome.

In our study of 54 patients evaluated after one year, 53 had excellent clinical results as assessed by the DASH questionnaire and 1 patient exhibited a poor clinical outcome, similar to other studies using this questionnaire [15].

According to the literature, the failure rate of conservative treatment ranges from 4.4 % to 31 % [20–23]. The most common complications are pain. aesthetic complaints, numbness, and loss of strength and function. The rate of 16.6 % complications in this study agrees with the values mentioned above.

Moreover, as there are major differences between the studies discussed, when considering patient characteristics such as those used in our research it can assist in choosing treatment and predicting the prognosis of this type of injury. Nevertheless, factors such as energy of the trauma and functional demands at work did not affect the results here.

Limitations of this study include the small number of patients with shortening greater than 20 mm and the use of subjective questionnaires rather than objective measurements such as strength and range of motion. Another limitation is the follow-up time of only one year. A longer follow-up period might expose deteriorating function, especially in patients with high-energy demand occupations. Other limitation is that anteroposterior ragiographs to measure clavicle length may cause some errors due to rotation failure.

## Conclusions

We conclude that the shortening of the clavicle that results from conservative treatment with a figure-of-eight bandage, even when more than 2 cm, does not affect subjective limb function.
